# Prevalence and factors associated with the death of older people hospitalized due to Covid-19 in the state of Paraná*

**DOI:** 10.1590/1980-220X-REEUSP-2023-0036en

**Published:** 2024-01-22

**Authors:** Luiz Hiroshi Inoue, Wanessa Cristina Baccon, Giovanna Brichi Pesce, Natan David Pereira, Isabela Vanessa Tavares Cordeiro Silva, Maria Aparecida Salci, João Ricardo Nickenig Vissoci, Luiz Augusto Facchini, Lígia Carreira

**Affiliations:** 1 Universidade Estadual de Maringá, Maringá, PR, Brazil.; 2 Duke University, Durham, Carolina do Norte, USA.; 3 Universidade Federal de Pelotas, Pelotas, RS, Brazil.

**Keywords:** Aged, Hospitalization, Mortality, Coronavirus, Nursing, Anciano, Hospitalización, Mortalidad, Coronavirus, Enfermería, Idoso, Hospitalização, Mortalidade, Coronavirus, Enfermagem

## Abstract

**Objective:**

To estimate the prevalence and to analyze the factors associated with the death of older people hospitalized due to Covid-19 in the state of Paraná.

**Method:**

Cross-sectional study conducted with secondary data from older people with a positive diagnosis of Covid-19 living in the State of Paraná, collected from March 1, 2020 to August 31, 2021. Prevalence ratios were obtained by adjusting the regression model.

**Results:**

A total of 16,153 deaths of older people hospitalized in the State of Paraná were analyzed. The adjusted model revealed an association between death and some factors such as: belonging to the age group of 75 to 84 years (PR = 1.28; CI_95%_ = 1.24–1.32) and 85 years or over (PR = 1.52; CI_95%_ = 1.45–1.59); male (PR = 1.17; CI_95%_ = 1.13–1.21); obesity (PR = 1.23; CI_95%_ = 1.16–1.29); other morbidities (PR = 1.25; CI_95%_ = 1.20–1.30); and having used ventilatory support (PR = 2.60; CI_95%_ = 2.33–2.86). Older people vaccinated against influenza had a probability of death reduced by 11% (PR = 0.89; CI_95%_ = 0.86–0.93).

**Conclusion:**

The association of age, sex, and diagnosis of previous comorbidities with unfavorable outcomes from Covid-19 was identified. Having received the flu vaccine provided protection to elderly people who contracted SARS-CoV-2.

## RESUMO

**Objetivo:**

Estimar a prevalência e analisar os fatores associados ao óbito de idosos hospitalizados por Covid-19 no Estado do Paraná.

**Método:**

Estudo transversal conduzido com dados secundários de idosos com diagnóstico positivo de Covid-19 residentes no Estado do Paraná, coletados no período de 01 de março de 2020 a 31 de agosto de 2021. As razões de prevalências foram obtidas por meio do ajuste de modelo de regressão.

**Resultados:**

Foram analisados 16.153 óbitos de idosos hospitalizados no Estado do Paraná. O modelo ajustado revelou associação do óbito a alguns fatores como: pertencer a faixa etária dos 75 a 84 anos (RP = 1,28; IC_95%_ = 1,24–1,32) e 85 anos ou mais (RP = 1,52; IC_95%_ = 1,45–1,59); sexo masculino (RP = 1,17; IC_95%_ = 1,13–1,21); obesidade (RP = 1,23; IC_95%_ = 1,16–1,29); outras morbidades (RP = 1,25; IC_95%_ = 1,20–1,30); e ter utilizado suporte ventilatório (RP = 2,60; IC_95%_ = 2,33-2,86). Idosos vacinados contra a gripe tiveram probabilidade de morte reduzida em 11% (RP = 0,89; IC_95%_ = 0,86–0,93).

**Conclusão:**

Identificou-se a associação da idade, sexo e diagnóstico de comorbidades prévias aos desfechos desfavoráveis da Covid-19. Ter recebido o imunizante contra a gripe conferiu proteção aos idosos que contraíram o SARS-CoV-2.

## RESUMEN

**Objetivo:**

Prevalencia y factores asociados a la muerte de personas mayores hospitalizadas por Covid-19 en el estado de Paraná*

**Método:**

Estudio transversal realizado con datos secundarios de personas mayores con diagnóstico positivo de Covid-19 residentes en el Estado de Paraná, recolectados del 1 de marzo de 2020 al 31 de agosto de 2021. Las razones de prevalencia se obtuvieron ajustando el modelo de regresión.

**Resultados:**

Se analizaron 16.153 muertes de personas mayores hospitalizadas en el Estado de Paraná. El modelo ajustado reveló asociación entre la muerte y algunos factores como: pertenecer al grupo etario de 75 a 84 años (RP = 1,28; IC_95%_ = 1,24–1,32) y 85 años o más (RP = 1,52; IC_95%_ = 1,45–1,59); masculino (RP = 1,17; IC_95%_ = 1,13–1,21); obesidad (RP = 1,23; IC_95%_ = 1,16–1,29); otras morbilidades (RP = 1,25; IC_95%_ = 1,20–1,30); y haber utilizado soporte ventilatorio (RP = 2,60; IC_95%_ = 2,33–2,86). Las personas mayores vacunadas contra la influenza tuvieron una probabilidad de muerte reducida en un 11% (RP = 0,89; IC_95%_ = 0,86–0,93).

**Conclusión:**

Se identificó la asociación de la edad, el sexo y el diagnóstico de comorbilidades previas con resultados desfavorables por Covid-19. Haber recibido la vacuna contra la gripe brindó protección a las personas mayores que contrajeron el SARS-CoV-2.

## INTRODUCTION

Covid-19 is an infectious disease caused by a variation of the coronavirus called SARS-CoV-2^(1)^. Several symptoms were observed; however, the most common were fever, fatigue, and cough^(2)^. It has a high potential for virulence and transmission, characteristics that contribute to its dissemination, serious outcomes, and death^(3)^.

Data from June 2023 revealed that 6,945,714 people died from complications from Covid-19 worldwide^(4)^. Of these, some groups were identified as more vulnerable to the disease, with the older population being one of the most affected, reaching 86% of all cases and with a probability of death 23 times higher, when compared to younger people^(5)^. In Brazil, in June of this same year, the number of deaths due to complications from Covid-19 exceeded 703 thousand^(6)^. Furthermore, in the State of Paraná, in the same period, 46,168 deaths from the disease were reported, with 30,287 cases in people aged 60 or over^(7)^.

According to the literature, some of the main factors associated with the worst outcomes from Covid-19 are age and previous diagnosis of diseases. In this context, individuals belonging to more advanced age groups are subject to changes in physiological functions, making them more susceptible to contracting the Covid-19 virus^(8)^.

Other relevant aspects for the high death rate in older victims of Covid-19 are the social and structural determinants of health. The association of these determinants with the health-disease process can answer numerous questions that define the profile of Covid-19 victims^(9)^. These collected and analyzed data can serve as guidance for prevention and public health promotion measures^(10)^.

Investigating the behavior of Covid-19 in the older population allows us to identify which characteristics were associated with death and how the manifestations may represent a decrease in quality of life and other health problems in the short and long term, a case known as Long Covid^(11,12)^. Furthermore, they allow health managers to implement public policies for monitoring the older people and, concomitantly, improve the quality of the assistance provided, so that it is increasingly resolute and assertive.

Therefore, considering the susceptibility of elderly people to serious outcomes from the disease, the hypothesis that sociodemographic and clinical factors may be associated with death from Covid-19 emerged. Thus, the objective of this study was to estimate the prevalence and to analyze the factors associated with the death of older people hospitalized due to Covid-19 in the state of Paraná.

## METHOD

### Design of Study

Cross-sectional study, based on secondary data in the public domain, on the deaths of older people hospitalized with Covid-19. We used the tool Strengthening the Reporting of Observational Studies in Epidemiology (STROBE)^(13)^.

### Local

The study was conducted in the state of Paraná, which, according to data from the Brazilian Institute of Geography and Statistics (IBGE) from 2020, has an estimated population of 11,675,661 inhabitants and, of these, 1,927,286 are older people^(14)^.

### Population, Selection Criteria, and Sample

During the period, 71,605 cases of Severe Acute Respiratory Syndrome (SARS) were reported among residents in the State of Paraná, between March 1, 2020 and August 31, 2021. Residence in the state was considered aiming at eliminating cases reported in Paraná, but whose residence was in other states. From this population, individuals considered older were selected, that is, those aged 60 years or over as provided in the first article of the Statute of the Elderly, Law 10.741/2003, who progressed to Severe Acute Respiratory Syndrome (SARS) due to Covid-19 and required hospitalization, both in the ward and ICU, totaling 45,505 cases. The exclusion of cases with missing information, and of those asymptomatic cases that were hospitalized, resulted in a sample consisting of 35,580 older people.

The sample only considered the State of Paraná due to its link to a cohort study, with a baseline anchored in the SIVEP-Gripe and Notifica Covid systems^(12)^. Thus, the data used for this research came from the database of the Paraná State Health Department (SESA-PR), with population identification and characterization data, allowing greater assertiveness in the identification of deaths and their associated factors.

Considering the large sample size and that the sample comprises hospitalized older people, reports of asymptomatic patients (2.8%) were excluded. Missing information regarding Flu Vaccination, Morbidities and occurrence of Symptoms were recognized as non-occurrence of events. The justification for this treatment is the filling practice observed by researchers during the analyzed period of 18 months of pandemic, in which due to the large volume of consultations and overload of health teams, priority was given to filling out positive responses. Therefore, variables with a large percentage of missing information were not considered for the univariate and multivariate analyses, so that they did not influence the study findings.

## VARIABLES OF INTEREST

### Dependent Variable

The dependent variable was defined as whether or not death from Covid-19 occurred during the hospitalization period. For determination, filling in the indicative field present in the occurrence notification form was considered.

### Independent Variable

The independent variables were distributed into two main groups: sociodemographic variables and clinical variables. For sociodemographic characteristics, the following were considered: Age group (60 to 74; 74 to 84; 85 years or more), Sex (female; male), Race/Color (white; black; yellow; indigenous), Residence in the same municipality of hospitalization (yes; no).

Clinical variables included: Vaccine against flu (yes; no), Diabetes (yes; no); Heart disease (yes; no); Obesity (yes; no); Other Morbidities (yes; no); Classification of Symptoms (Common/Mild; Moderate/Severe); and Use of Ventilatory Support (yes; no).

Furthermore, groups such as the following were considered: 1) Other Morbidities (comprises immunodeficiency, asthma, chronic lung disease, chronic neurological disease, and chronic kidney disease); 2) Common/Mild Symptoms (which include: cough, sore throat, abdominal pain, fatigue, loss of smell, and loss of taste); and 3) Moderate/Severe Symptoms (include fever, saturation <95%, diarrhea, vomiting, dyspnea, and respiratory distress), following the same presentation as the notification form used. In this context, the variable Use of Ventilatory Support considered both invasive devices (orotracheal tube) and non-invasive devices (oxygen catheter, Venturi mask).

### Data Collection

Data were collected from individual registration forms of cases of Severe Acute Respiratory Syndrome (SARS) from the SIVEP-Gripe platform, an Epidemiological Surveillance System of the Health Surveillance Secretariat (SVS) for recording cases of deaths due to SARS^(15)^. The period from March 1, 2020 to August 31, 2021 was chosen with the aim of analyzing the most recent data available at the time of collection.

### Data Analysis and Treatment

The data were organized in electronic spreadsheets and processed in the statistical software R, version 4.0.4, using the packages *dplyr* and *epitools*. A confidence level of 95% was considered in all analyses performed.

For the descriptive analysis of the data, absolute and relative frequencies of the variables were estimated based on the outcome, applying Pearson’s Chi-square test to compare the variables. The analysis of associated factors was performed using Quasi-Poisson regression models. This distribution was used due to the breach of the equidispersion assumption for the Poisson distribution, which indicates equal mean and variance. Gross and adjusted models were estimated to identify associations and estimate prevalence ratios as follows:

(1) The raw models were calculated using Pearson’s Chi-square test, using the unconditional maximum likelihood method. To construct the multiple model, p value < 0.20 was defined as the cutoff point in the raw model;
(2) The adjusted models were estimated using Quasi-Poisson regression with robust variance, to control variance overdispersion. Therefore, to construct the adjusted model, the stepwise forward method, which tests variables by significance level from lowest to highest value, was used.

Model adjustment was tested by estimating the parameters: (1) Akaike Information Criterion (AIC), with modulation for the Quasi-Poisson distribution (Quasi AIC); (2) Bayesian Information Criterion (BIC), with modulation for the Quasi-Poisson distribution (Quasi BIC); and (3) McFadden’s Pseudo R^2^. The best model was considered to be the one presenting the lowest Quasi AIC, lowest Quasi BIC, and highest Pseudo R^2^. The variables that presented p < 0.05 remained in the final model.

### Ethical Aspects

The study obtained a favorable opinion from the Research Ethics Committee of the State University of Maringá under opinion no. 4.165.272 and CAAE: 34787020.0.0000.0104. Furthermore, it received approval from the Health Department of the State of Paraná, represented by Hospital do Trabalhador, obtaining opinion no. 4.214.589 and CAAE: 34787020.0.3001.5225, on August 15, 2020.

## RESULTS

The sample analyzed consisted of 35,580 records of older people, individuals aged 60 or over, with a confirmed diagnosis of Severe Acute Respiratory Syndrome (SARS) due to Covid-19, residents and hospitalized in the state of Paraná. The number of older people who died was 16,153, which corresponds to approximately 45% of the total sample. In the selected sample, the median age is 71 years with the first and third quartiles equal to 65 and 78 years, respectively, resulting in an interquartile range of 13 years. Furthermore, the maximum age observed was 113 years.

Taking into account the categorization of ages into age groups, we have: 22,621 (63.6%) older people between 60 and 74 years old, 9,192 (25.8%) older people between 75 and 84 years old, and 3,767 (10.5%) older people with 85 years or older. The findings show that, although the number of hospitalizations was higher in the age group of 60 to 74 years, the prevalence of deaths was proportionally similar in the more advanced age group, reinforcing that the severity of the disease is increased concomitantly with the increase of age. Regarding sex, older males had a 6.1% higher percentage of deaths compared to females. Regarding race/color, there was a higher prevalence of death in elderly people self-declared as black, indigenous/yellow (47.3%). Moreover, the prevalence of deaths among elderly people not residing in the city of hospitalization was 5.5% higher when compared to individuals residing in the city of hospitalization (Table 1).

**Table 1:** Sociodemographic and clinical characteristics of older people hospitalized for Covid-19 who died in the State of Paraná, Brazil, 2021 (n = 35.580) – Maringá, PR, Brazil, 2023.

Variables	Case Progression						p-value
	Hospitalized (n = 35,580)		Hospital discharge (n = 19,427)		Deaths (n = 16,153)		
	n	%	n	%	n	%	
***Sociodemographic***							
**Age range**							<0.0001
60 to 74	22,621	63.6	13,543	59.9	9,078	40.1	
75 a 84	9,192	25.8	4,424	48.1	4,768	51.9	
85 or over	3,767	10.6	1,460	38.8	2,307	61.2	
**Sex**							<0.0001
Female	16.582	46.6	9.595	57.9	6.987	42.1	
Male	18.998	53.4	9.832	51.8	9.166	48.2	
**Race/color**							<0.0001
White	29.804	83.8	16.381	55.0	13.423	45.0	
Black	5.281	14.8	2.785	52.7	2.496	47.3	
Indigenous/Yellow	495	1.4	261	52.7	234	47.3	
**Lives in the city of hospitalization**							<0.0001
Yes	23.412	65.8	13.221	56.5	10.191	43.5	
No	12.168	34.2	6.206	51.0	5.962	49.0	
***Clinical***							
**Flu vaccine**							<0.0001
Yes	5.840	16.4	3.531	60.5	2.309	39.5	
No	29.740	83.6	15.896	53.4	13.844	46.6	
**Diabetes**							
Yes	11.125	31.3	5.681	51.1	5.444	48.9	<0.0001
No	24.455	68.7	13.746	56.2	10.709	43.8	
**Heart diseases**							
Yes	17.012	47.8	8.739	51.4	8.273	48.6	<0.0001
No	18.568	52.2	10.688	57.6	7.880	42.4	
**Obesity**							
Yes	2.862	8.0	1.307	45.7	1.555	54.3	<0.0001
No	32.718	92.0	18.120	55.4	14.598	44.6	
Other Morbidities*							
Yes	6.837	19.2	2.880	42.1	3.957	57.9	<0.0001
No	28.743	80.8	16.547	57.6	12.196	42.4	
**Symptoms**							
Common/Mild^†^	1.378	3.9	921	66.8	457	33.2	<0.0001
Moderate/Severe^‡^	34.202	96.1	18.506	54.1	15.696	45.9	
**Use of Ventilatory Support**							
Yes	29.337	82.5	14.366	49.0	14.971	51.0	<0.0001
No	6,243	17.5	5,061	81.1	1,182	18.9	

*Immunodeficiency; asthma; other chronic lung disease; chronic neurological disease; chronic kidney disease. ^†^Cough; sore throat; abdominal pain; fatigue; loss of smell; loss of taste. ^‡^Fever; O2 saturation <95%; diarrhea; vomiting; dyspnea; respiratory discomfort.

The prevalence of deaths in older people who previously received the flu vaccine was 7.1% lower when compared to those who were not vaccinated. Older people with pre-existing comorbidities such as diabetes, heart disease, obesity, and other morbidities had a death rate that was approximately 15% higher compared to those without previous diagnoses. Furthermore, those who presented symptoms considered moderate/severe had a 12.7% higher death rate compared to those who presented common/mild symptoms. Older people who required the use of ventilatory support had a 32.1% higher death rate than those who did not use any devices.

In the raw analysis, with the exception of the variable race/indigenous color/yellow (p < 0.3215), most variables presented significant statistical results associated with the death of hospitalized older people (p < 0.05). A linear increase in the prevalence of deaths was observed with age group and a decrease in relation to older people vaccinated against influenza with a protective factor (PR = 0.85; CI_95%_ = 0.82-0.88). In relation to sex, the male population had a 15% higher probability of death (IC_95%_ = 1.12–1.17) compared to females (Table 2).

**Table 2:** Raw model for associations between sociodemographic and clinical characteristics of hospitalized older people. Paraná, Brazil, 2021 (n = 16,153) – Maringá, PR, Brazil, 2023.

Variables		Death (n = 16,153)		Raw Model		p-value
		n	%	PR	CI 95%	
***Sociodemographic***						
**Age range**						
60 to 74		9,078	40.1	Reference	–	–
75 a 84		4,768	51.9	1.29	1.26–1.33	<0.0001
85 or over		2,307	61.2	1.53	1.48–1.57	0.0010
**Sex**						
Female		6,987	42.1	Reference	–	–
Male		9,166	48.2	1.15	1.12–1.17	<0.0001
**Race/color**						
White		13,423	45.0	Reference	–	–
Black		2,496	47.3	1.05	1.02–1.08	0.0027
Indigenous/Yellow		234	47.3	1.05	0.96–1.15	0.3215
**Lives in the city of hospitalization**						
Yes		10,191	43.5	Reference	–	–
No		5,962	49.0	1.13	1.10–1.15	<0.0001
***Clinical***						
**Flu vaccine**						
No		13,844	46.6	Reference	–	–
Yes		2,309	39.5	0.85	0.82–0.88	<0.0001
**Diabetes**						
No		10,709	43.8	Reference	–	–
Yes		5,444	48.9	1.12	1.09–1.14	<0.0001
**Heart diseases**						
No		7,880	42.4	Reference	–	–
Yes		8,273	48.6	1.15	1.12–1.17	<0.0001
**Obesity**						
No		14,598	44.6	Reference	–	–
Yes		1,555	54.3	1.21	1.18–1.26	<0.0001
Other Morbidities*						
No		12,196	42.4	Reference	–	–
Sim		3,957	57.9	1.36	1.33–1.40	<0.0001
**Symptoms**						
Common/Mild^†^		457	33.2	Reference	–	–
Moderate/Severe^‡^		15,696	45.9	1.38	1.28–1.49	<0.0001
**Use of Ventilatory Support**						
No		1,182	18.9	Reference	–	–
Yes		14,971	51.0	2.70	2.56–2.84	<0.0001

*Immunodeficiency; asthma; other chronic lung disease; chronic neurological disease; chronic kidney disease. ^†^Cough; sore throat; abdominal pain; fatigue; loss of smell; loss of taste. ^‡^Fever; O2 saturation <95%; diarrhea; vomiting; dyspnea; respiratory discomfort.

After adjusted analysis, the linear increase in the prevalence of deaths with age remained and the age group 85 years or more was 1.5 times higher compared to 60 to 74 years. The probability of older men dying from Covid-19 was 1.17 times greater compared to women. Being vaccinated against the flu reduced the likelihood of death by 11% compared to unvaccinated older people. Older people with diabetes and obesity had a 1.09 and 1.23 times higher probability of death respectively compared to those without the diseases. The variables race/color, residence in the city of hospitalization, heart disease and symptoms lost significance after analysis adjusted for variables in the same category. The associations between elderly people with other morbidities and death were maintained. The probability of death in elderly people who used ventilatory support was 2.6 times higher compared to those who did not use it (Table 3).

**Table 3:** Adjusted model controlled by the effect of the death variable for the association between sociodemographic and clinical variables of hospitalized older people. Paraná, Brazil, 2021 (n = 16,153) – Maringá, PR, Brazil, 2023.

Variables	Death (n = 16,153)		Adjusted Model		p-value
	n	%	PR	CI 95%	
***Sociodemographic***					
**Age range**					
60 to 74	9,078	40.1	Reference	–	–
75 a 84	4,768	51.9	1.28	1.24–1.32	<0.0001
85 or over	2,307	61.2	1.52	1.45–1.59	<0.0001
**Sex**					
Female	6,987	42.1	Reference	–	–
Male	9,166	48.2	1.17	1.13–1.21	<0.0001
***Clinical***					
**Flu vaccine**					
No	13,844	46.6	Reference	–	–
Yes	2,309	39.5	0.89	0.86–0.93	<0.0001
**Diabetes**					
No	10,709	43.8	Reference	–	–
Yes	5,444	48.9	1.09	1.06–1.13	<0.0001
**Obesity**					
No	14,598	44.6	Reference	–	–
Yes	1,555	54.3	1.23	1.16–1.29	<0.0001
Other Morbidities*					
No	12,196	42.4	Reference	–	–
Yes	3,957	57.9	1.25	1.20–1.30	<0.0001
**Use of Ventilatory Support**					
No	1,182	18.9	Reference	–	–
Yes	14,971	51.0	2.60	2.33–2.86	<0.0001

*Immunodeficiency; asthma; other chronic lung disease; chronic neurological disease; chronic kidney disease.

## DISCUSSION

In this study, our objective was to estimate the prevalence and to analyze the factors associated with the death of older people hospitalized due to Covid-19 in the state of Paraná. These findings revealed that the probability of an older person dying from Covid-19 gradually increases, depending on some factors to which the individual is conditioned. Advanced age, combined with comorbidities, means clinical worsening and a worse outcome of Covid-19 infection in this population, corroborating findings in the literature^(16)^. Furthermore, the resulting delay in confirming the diagnosis can influence the progression of the disease, increasing the possibility of developing serious outcomes and death^(17)^.

Some predictors of worse Covid-19 prognoses are highlighted in the literature, the most common being advanced age and the diagnosis of comorbidities. The association of these factors means, for this audience, the possibility of occurrence of hyper-inflammatory responses (leukocytosis, neutrophilia and elevated levels of C-reactive protein and ferritin), as well as organic and coagulation dysfunctions (increased plasma glucose, altered serum creatinine and lactate dehydrogenase and increased D-dimer levels)^(2,18)^.

These inflammatory markers and organ dysfunctions, observed in elderly patients with Covid-19, are closely related to age-associated dysregulation of the immune system. “Inflammaging” plays an important role in the pathogenesis of Covid-19, resulting in severe clinical conditions, especially severe lung damage^(19)^.

In this regard, with aging there is an increase in pro-inflammatory cytokines, including IL-6, TNFα and NLRP3 inflammasome, which are associated with age-related diseases such as autoimmune diseases, cancer, and metabolic disorders. These cytokines contribute to chronic inflammation and tissue dysfunction. Furthermore, other inflammatory biomarkers, such as IL-18, IL-1β, CRP, and IL-15, also increase with age. In addition, in the immune system’s attempt to maintain homeostasis, there is also an increase in anti-inflammatory molecules, such as IL-10 and TGF-β, which play a role in the immune system’s attempt to maintain homeostasis. The balance between pro-inflammatory and anti-inflammatory cytokines plays a determining role in the development of age-related diseases, such as coronary heart disease, osteoarthritis, and neurodegenerative diseases^(20)^.

Still regarding this aspect, the destabilization of the immune system of older people, during the Covid-19 pandemic, was enhanced by social isolation and the consequent reduction or lack of regular physical activity^(21)^.

A study carried out in Southern Africa showed that leukocytosis and neutrophilia are diseases highly associated with the death of older people due to Covid-19, since the high count of leukocytes and neutrophils can cause cascades of inflammation with the production of inflammatory cytokines. This process is also associated with the progression to more serious conditions and death from Covid-19^(22)^.

It is important to highlight that older people who develop more severe conditions can develop sequelae that affect physical and cognitive functions in the long term, a condition called Long Covid. In this context, even after the end of the acute phase of the disease (12 weeks), individuals may develop symptoms and signs not associated with other diagnoses, meaning a late manifestation of Covid-19^(23)^.

Another important factor to be considered is the greater susceptibility to infection and complications from Covid-19 in older men. A meta-analysis study pointed to the possibility of sexual hormones acting on innate and adaptive immunity, contributing to lower numbers of infection and death from Covid-19 in women. Furthermore, the same study refers to the possibility that a greater susceptibility of males to the disease is related to behavioral factors that contribute to the development of comorbidities and greater exposure of men to the virus^(16)^.

Regarding previous diagnoses, it is clear that older people diagnosed with Diabetes Mellitus (DM) and obesity are more susceptible to developing the severe form of the disease and dying, the same unfavorable scenario evidenced during other viral pandemics, such as Severe Acute Respiratory Syndrome (SARS), in 2003, Influenza (H1N1), in 2009, and Respiratory Syndrome of the Middle East, in 2012^(24)^.

Other pre-existing diseases, such as asthma and lung diseases, also increase the likelihood of death from Covid-19 among older people^(25)^. In Mexico, a study showed that the presence of respiratory pathologies increases the risk of death when compared to individuals without a diagnosis of these diseases^(24)^.

In this context, elderly people with neurological diseases may also have an increased risk of death. In a study conducted in England, older people with neurological diseases, as well as other pre-existing comorbidities, such as kidney disease and heart disease, had their chances of death increased by 150%^(25)^.

The use of ventilatory support also has a strong association with the death of older people from Covid-19^(26,27)^. In the aging process, defense cells undergo several changes, destabilizing the immune system and making the older person more susceptible to widespread complications and, consequently, increasing the chances of needing respiratory support in more serious cases of the disease^(28)^.

Regarding the flu vaccine, a study carried out in the USA indicated that, in accordance with the increase in vaccination coverage, there was a decrease in mortality in the elderly due to Covid-19^(29)^, a result similar to the findings of the present study.

The rapid spread of Covid-19 around the world showed the unpreparedness of health systems to face the pandemic of new diseases. The rapid spread of SARS-CoV-2 and the rampant dissemination of the infection among more vulnerable groups, such as older people, resulted in the overload of health systems in caring for this population. Furthermore, this event allows the reflection that health systems must carry out an adaptation process to monitor previous morbidities existing in this already vulnerable public, but also new diagnosed cases of Covid-19 and Long Covid, meaning an increase in demand of health care provided to this group^(30)^.

As limitations of the present study, it should be noted that, as the information is available in secondary databases and originating from administrative health systems, there may be underreporting and incomplete information on some variables. However, the selection of the analysis method was made in such a way not to allow the interference of missing data on other variables and to ensure greater assertiveness in health statements. Also, the evaluation limited to hospitalized patients may have overestimated the association between the factors and death from Covid-19, due to the hospital nature of the study, where older people are more likely to present severity and comorbidities, and cases of the disease tend to be moderate or severe. Even with the aforementioned limitations, the findings of this study allowed the evaluation of epidemiological and clinical characteristics of coronavirus infection in the State of Paraná.

Furthermore, it should be highlighted that carrying out studies with this methodological nature encourages the conduct of similar research in other regions, with the aim of allowing an understanding of the real influence on local conditions and the behavior of the disease in different population strata, highlighting the importance of producing and disseminating science that is applicable and beneficial to the population.

Regarding this aspect, the research findings still serve as a framework for health managers to develop and implement public policies based on science, founded on a real understanding of the scenario experienced by the population, based on all its weaknesses, so that the assistance provided becomes increasingly resolute and ensures greater assertiveness in health actions.

## CONCLUSION

It was evident that some factors were related to death from Covid-19 in the elderly in the State of Paraná. Higher age groups, being male, having a diagnosis of DM, obesity or other morbidities, as well as having used ventilatory support during the period of hospitalization for the disease, meant greater chance of severity and death from Covid-19.

Administration of the flu vaccine has been shown to be beneficial in providing protection to older people who have been exposed to SARS-CoV-2. This correlation highlights the relevance of preventive measures, such as immunization, mitigation of the negative impacts of the pandemic on a more vulnerable population, reinforcing the continued importance of vaccination campaigns to ensure older people’s health and well-being.

It is recommended that current public policies be improved in terms of effectiveness and efficiency. It is expected that the study will provide support for improvement in order to effectively meet the needs of the older population. This includes promoting a better quality of life, ensuring access to health, education, and income opportunities, with the aim of reducing the vulnerability of older people to Covid-19.
